# Breaking the cycle: immune complexes, complement activation, and novel immunotherapies in lupus nephritis

**DOI:** 10.3389/fimmu.2025.1624850

**Published:** 2025-10-09

**Authors:** Cuiting Dong, Mingda Wu, Haonan Liu, Kunpeng Yang, Shuang Ma, Yan Guo, Yuejiao Lan, Xiaodan Lu

**Affiliations:** ^1^ Changchun University of Chinese Medicine, Changchun, Jilin, China; ^2^ Jilin Province People’s Hospital, Changchun, Jilin, China

**Keywords:** lupus nephritis, biomarkers, immunotherapy, podocyte injury, targeted therapy, systemic lupus erythematosus

## Abstract

Lupus nephritis (LN), a severe manifestation of systemic lupus erythematosus (SLE), is driven by immune complex deposition and complement activation, resulting in glomerular inflammation and podocyte injury. Beyond being passive targets, podocytes actively modulate renal immunity through cytokine secretion and antigen presentation. Recent advances in urinary biomarkers such as NGAL, TWEAK, and MCP-1 and composite indices like the Renal Activity Index for Lupus (RAIL) offer dynamic and noninvasive monitoring of disease activity. Immunotherapy has transitioned from nonspecific immunosuppression to targeted biologics, with agents such as belimumab and telitacicept improving outcomes by modulating B-cell function. Additionally, emerging therapies including bortezomib and daratumumab demonstrate efficacy in refractory LN through plasma cell depletion. This review summarizes current immunological insights, biomarker innovations, and immunotherapy strategy to support precision medicine and improve long-term renal prognosis in LN.

## 1 Introduction

Lupus nephritis (LN), one of the most common and severe manifestations of systemic lupus erythematosus (SLE), results from immune complex (IC) deposition in the kidneys (1, 2). SLE is a chronic, multisystem autoimmune disease triggered by genetic and environmental factors—including hormonal imbalances, infections, and drug exposures—that disrupt immune tolerance and promote autoantibody production (3, 4). Approximately 50% of SLE patients develop LN (5), characterized by glomerular accumulation of autoantibodies, such as anti-dsDNA, which activate the complement cascade, recruit inflammatory cells, and injure podocytes (6, 7). Rather than serving only as passive targets, podocytes actively contribute to disease by expressing pattern recognition receptors, secreting cytokines, and presenting antigens that amplify the immune responses and injury (8, 9).

Despite renal biopsy being the definitive method for LN histopathological assessment, its invasiveness limits repeated use in routine clinical management (10). Current therapies induce complete renal response in only approximately 20–40% of patients, and 20% progress to end-stage kidney disease (ESKD) within five years (11), underscoring the need for improved biomarkers and treatments. Urinary biomarkers, including NGAL and TWEAK, offer potential for disease monitoring (12), while biologics such as belimumab and telitacicept target B-cell signaling pathways with increasing success in clinical trials (13–15). This review integrates recent insights into LN pathogenesis, diagnostic biomarkers, and therapeutic innovations to support personalized care and research progress.

## 2 Pathogenesis of lupus nephritis

The development of LN occurs within the genetic framework of SLE, with its increased prevalence highlighting the critical influence of epigenetic modifications (16). The disease process involves a complex breakdown of immune regulation, triggered by viral infections, environmental pollutants, and drug-induced effects (17–20). This cascade begins with impaired removal of apoptotic cells, resulting in the abnormal release of nuclear antigens. Unprocessed nucleic acids and nuclear proteins stimulate B-cell activation through Toll-like receptor (TLR)-dependent pathways, promoting the production of anti-nuclear antibodies (21). These antibodies contribute to the formation of circulating immune complexes (CICs) or, via molecular mimicry, generate *in situ* complexes that target structural elements of the glomerular basement membrane (22, 23). While CICs accumulate in renal tissues due to hemodynamic forces, *in situ* complexes directly bind glomerular antigens (24, 25). Both mechanisms ultimately trigger complement activation and neutrophil recruitment (26). Concurrently, immune complexes induce glomerular endothelial and mesangial cells to release proinflammatory cytokines, including monocyte chemoattractant protein-1 (MCP-1) and tumor necrosis factor-α (TNF-α) (27, 28). These mediators intensify inflammation via NF-κB signaling, culminating in podocyte damage and impairment of the glomerular filtration barrier (29).

## 3 Podocyte injury in lupus nephritis

### 3.1 Immune complex-mediated injury and complement activation

Podocyte injury in LN displays significant pathological diversity across different disease classes (30). In proliferative LN (classes III/IV), anti-dsDNA antibodies activate complement pathways and induce endothelial inflammation, while subendothelial immune complex (IC) accumulation leads to structural alterations in podocytes (6, 31). These changes include cytoskeletal reorganization through reduced nephrin expression and inhibited VEGF-A signaling (32). Conversely, class V membranous LN primarily involves subepithelial IC deposition, which stimulates localized complement activation and functional impairment of podocytes (33). This manifests as foot process effacement without basement membrane disruption, differing mechanistically from the structural damage (podocyte detachment, basement membrane rupture) seen in proliferative forms. These distinctions may account for the more favorable long-term outcomes observed in class V disease (34). Emerging evidence indicates that the extent of glomerular endothelial damage in proliferative LN positively associates with foot process width (FPW). Furthermore, IgG antibodies promote podocyte cytoskeletal reorganization by activating β1-integrin-dependent pathways (32). Dysregulation of the VEGF-endothelin axis also contributes to filtration barrier dysfunction through increased proinflammatory mediator release and impaired podocyte–endothelial crosstalk (35). Interestingly, while VEGF-A downregulation in proliferative LN contributes to endothelial injury and proteinuria, class V membranous LN often shows preserved or even upregulated VEGF-A levels, potentially reflecting a compensatory response to subepithelial immune complex deposition (36, 37). These subtype-specific differences underscore a knowledge gap in understanding VEGF signaling dynamics and highlight the need for tailored therapeutic strategies targeting angiogenic pathways.

### 3.2 Cytoskeletal disruption and signaling pathways

#### 3.2.1 Immune complex deposition

**Figure 1:**
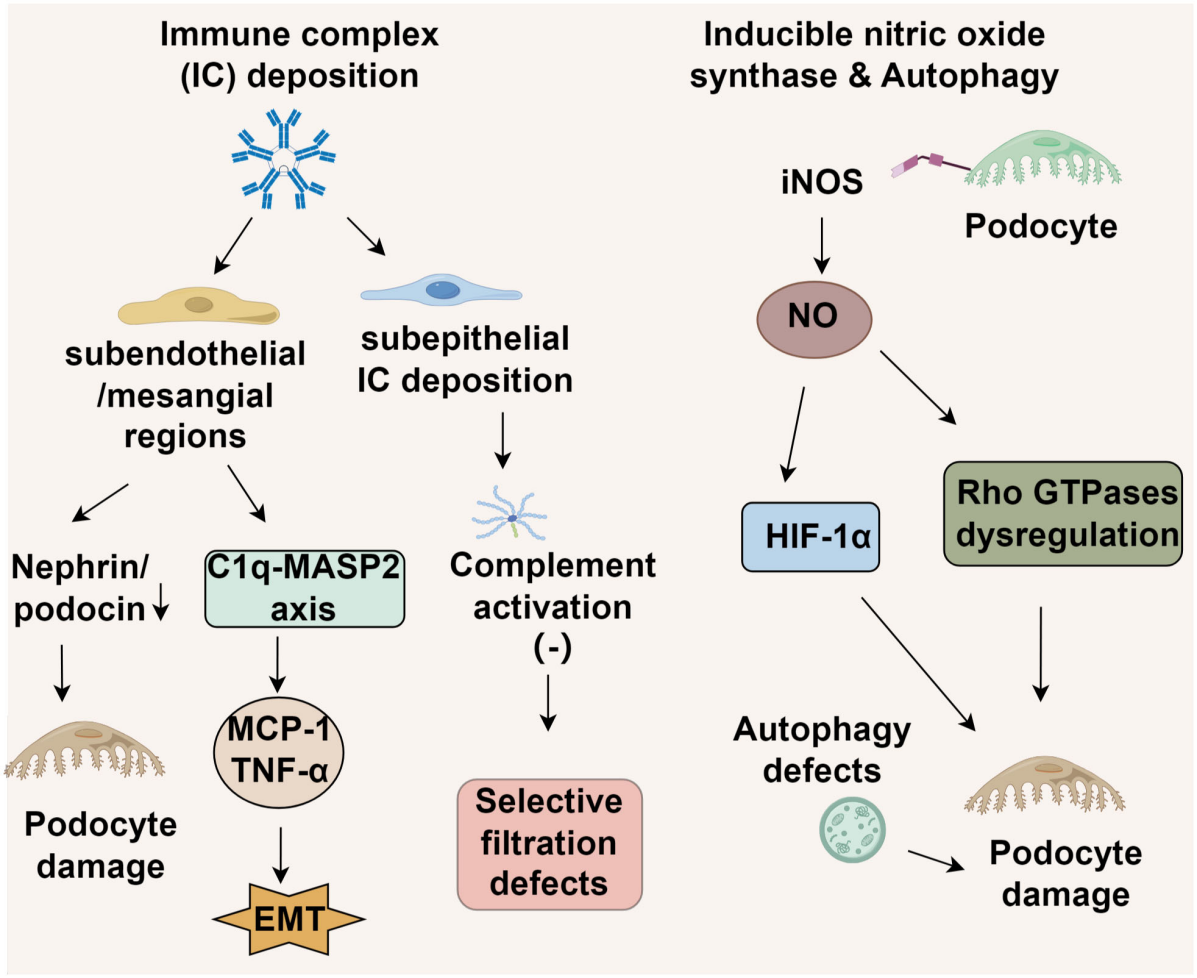
Molecular mechanisms of lupus nephritis podocyte injury.

The deposition patterns of ICs in LN vary significantly based on their anatomical distribution. In proliferative LN (classes III/IV), ICs predominantly accumulate in subendothelial and mesangial regions (38). This initiates podocyte injury through cytoskeletal destabilization, which is mediated by reduced expression of nephrin and podocin, and promotes endothelial-mesenchymal transition (6). These effects occur via Fcγ receptor-dependent complement activation and NF-κB-driven release of inflammatory cytokines such as MCP-1 and TNF-α (7). Conversely, class V membranous LN is distinguished by subepithelial IC deposition, where the hydrophobic properties of the glomerular basement membrane restrict complement activation, primarily involving C5b-9 membrane attack complexes (39, 40). This results in foot process effacement and internalization of slit diaphragm proteins, leading to selective filtration defects rather than extensive inflammatory damage (7, 41). Importantly, FPW expansion correlates with clinical markers of disease severity across LN subtypes. However, the underlying mechanisms differ. In class V LN, FPW alterations reflect charge-selective barrier impairment, whereas in proliferative LN, FPW abnormalities stem from structural damage to the lamina rara interna and podocyte detachment. These distinctions support the need for histotype-specific treatment approaches (41) (**Figure 1**).

Endothelial damage in LN demonstrates subtype-dependent characteristics (42). In proliferative LN (classes III/IV), excessive complement activation triggered by anti-dsDNA antibodies causes widening of the basement membrane-endothelial cleft and degradation of the endothelial glycocalyx (43). In contrast, class V LN features endothelial-mesenchymal transition (EndMT) induced by podocyte-derived TGF-β overexpression, leading to basement membrane thickening while largely sparing endothelial integrity (42). Notably, genetic deficiencies in complement regulatory proteins or autoantibodies targeting complement factors can perpetuate alternative pathway activation. This promotes podocyte dysfunction and disrupts the thrombomodulin-protein C system, potentially precipitating aHUS-like pathology and identifying a distinct molecular subset of nephrotic-range proteinuria (44).

#### 3.2.2 iNOS-mediated podocyte injury in lupus nephritis

Emerging evidence implicates podocyte-specific upregulation of inducible nitric oxide synthase (iNOS) as a key mediator of structural damage in lupus nephritis (45), though its exact pathogenic role remains to be fully elucidated. The current paradigm suggests that excessive nitric oxide production leads to podocyte injury through hypoxia-inducible factor 1α (HIF-1α) activation and subsequent dysregulation of Rho GTPases, particularly Cdc42 and Rac1 (46). This dysregulation disrupts podocyte structural integrity, highlighting iNOS and its downstream effectors as potential therapeutic targets to mitigate NO-induced podocyte injury and slow disease progression (46). Parallel studies have identified autophagy as a critical regulator of podocyte survival in LN. Early disease stages are characterized by distinct alterations in autophagy-related pathways, which may determine podocyte fate. *In vitro* models demonstrate that LN-like conditions upregulate cyclooxygenase-2 (COX-2) and induce endoplasmic reticulum (ER) stress in podocytes, mechanistically linked to activation of the unfolded protein response (UPR) transcription factor ATF4 (47). Pharmacological inhibition of COX-2 attenuates autophagy induction in these models, while ATF4 knockdown abolishes LN-associated COX-2 overexpression, suggesting a causal role for the ATF4-COX-2 axis in podocyte injury (47). Multiple studies have demonstrated a strong correlation between iNOS level and the progression of disease phenotypes in several murine LN models (48, 49). Notably, the iNOS inhibitor SD-3651 significantly ameliorates both proteinuria and podocytopathy in experimental LN mice (49). Despite promising preclinical data supporting iNOS inhibition, clinical translation faces challenges, including off-target effects of pan-iNOS inhibitors and the absence of reliable biomarkers for patient stratification. Future therapeutic strategies require selective iNOS modulation combined with autophagy restoration to preserve podocyte function in LN.

### 3.3 Podocyte-related biomarkers in lupus nephritis

Studies on podocyte-linked biomarkers in LN highlight their dual function as both immune complex-mediated injury targets and disease progression markers. The reduced expression of podocyte-specific proteins in glomeruli emphasizes the critical role of cytoskeletal disruption in LN glomerulopathy pathogenesis (50). Furthermore, urinary sediment analysis in active LN patients detects abnormal levels of podocalyxin, synaptopodin mRNA, and immature nephrin/GLEPP1 proteins, supporting their use as noninvasive diagnostic tools for glomerular damage (51). Proteomic analyses have identified α-enolase and annexin A1 as podocyte-targeted autoantigens in LN, where their strong interaction with glomerular IgG suggests involvement in immune complex formation (52, 53). Podocyte-derived microparticles (MPs), carrying surface markers like annexin V and podocalyxin, serve as dynamic indicators of renal disease activity and histopathological changes in SLE (54). Importantly, NF-κB signaling is consistently implicated in proinflammatory gene expression in LN podocytes, driving production of cytokines and apoptotic markers (55). Besides, the NF-κB pathway also orchestrates cell survival and tissue repair mechanisms. For instance, selective inhibition of canonical NF-κB mitigates glomerular inflammation, while blockade of noncanonical branches could disrupt podocyte adaptation and regeneration (29, 56). Future studies need to clarify which subunits and downstream effectors of NF-κB signaling represent optimal therapeutic windows in LN (57). The extent of podocyte injury closely parallels proteinuria severity, as research shows that foot process effacement (FPE) in both proliferative and non-proliferative LN correlates with significant protein loss, alongside diminished expression of mature podocyte markers like synaptopodin, nephrin, and GLEPP1 in proliferative forms (34). Additionally, these injured podocytes exacerbate LN-related inflammation by releasing cytokines such as IL-1β, TNF-α, IFN-α, and IFN-γ, which sustain renal damage (58). Osteopontin (OPN), produced by T cells, enhances macrophage recruitment into glomeruli (59) and may also modulate podocyte signaling and movement, thereby contributing to proteinuria onset and progression (60).

## 4 Noninvasive urinary biomarkers in lupus nephritis

**Figure 2:**
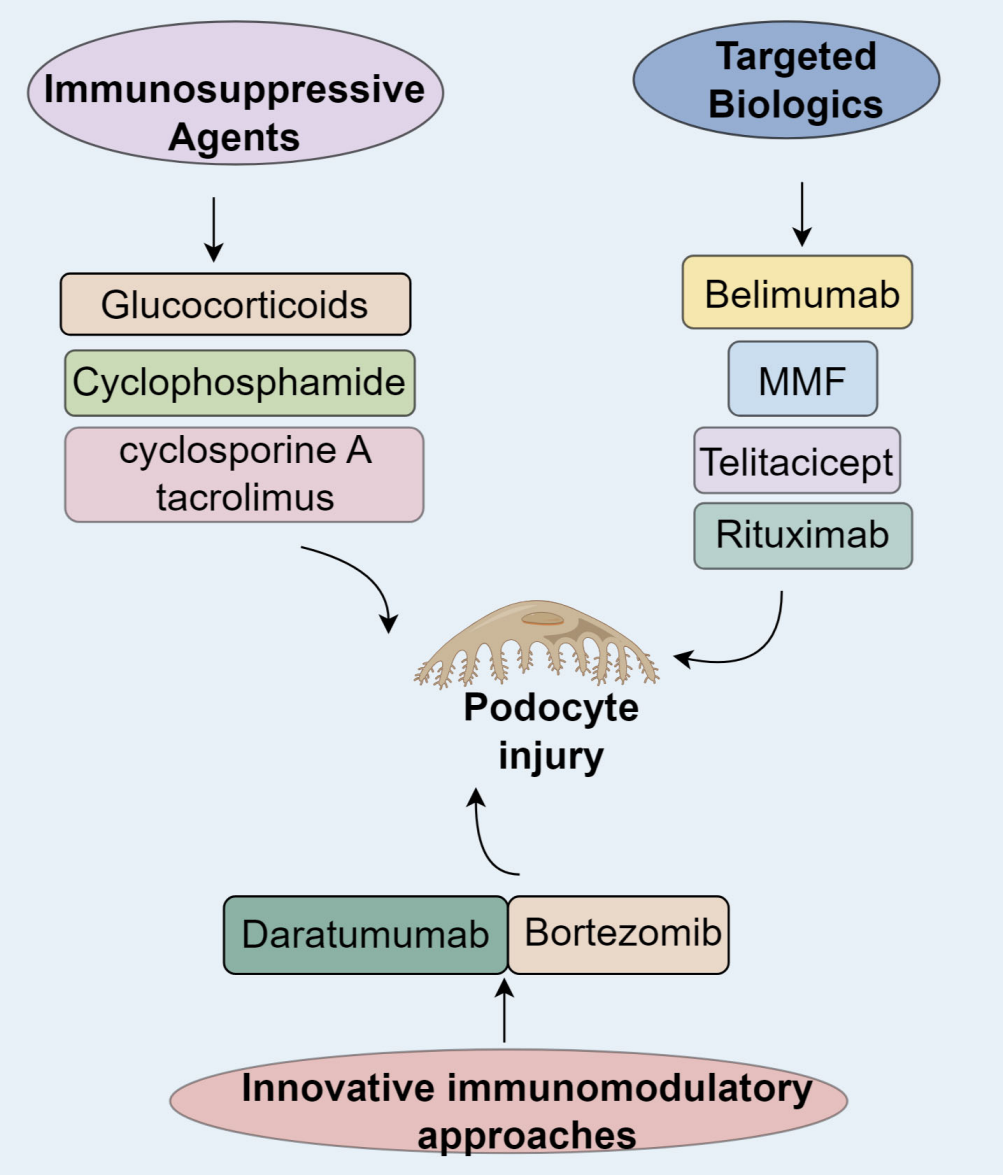
Immunotherapy in lupus nephritis.

Urinary biomarkers serve as crucial tools for the noninvasive assessment and longitudinal tracking of LN, offering insights into renal immune dysfunction and inflammatory damage (61, 62). Among individual biomarkers, neutrophil gelatinase-associated lipocalin (NGAL) demonstrates strong associations with disease severity and therapeutic efficacy (63). Similarly, tumor necrosis factor-related weak inducer of apoptosis (TWEAK) is implicated in NF-κB-mediated inflammatory pathways and LN-specific renal pathology (64). Monocyte chemoattractant protein-1 (MCP-1), a key chemokine, reflects macrophage accumulation and aids in histopathological classification, while vascular cell adhesion molecule-1 (VCAM-1) facilitates leukocyte migration and correlates with disease exacerbations (65–67). Multiparametric strategies, such as the Renal Activity Index for Lupus (RAIL), improve diagnostic precision by combining several biomarkers, such as NGAL, KIM-1, and MCP-1, to forecast treatment responses. Urinary proteomic profiling further enhances detection by identifying renal-specific pathological patterns, though its clinical implementation faces hurdles due to preanalytical inconsistencies and methodological disparities (68–71). Although standalone biomarkers exhibit limited discriminatory capacity, composite panels like RAIL show enhanced predictive value, highlighting their utility in real-time LN monitoring. Nevertheless, validation across diverse populations and standardization of analytical protocols are essential for widespread clinical adoption (**Figure 2**).

## 5 Immunotherapy in lupus nephritis

### 5.1 Traditional immunosuppressive agents

Immunosuppressive drugs exhibit podocyte-preserving properties in LN by modulating slit diaphragm components. Glucocorticoids reinforce the actin cytoskeleton, stimulate RhoA/ROCK pathways, and enhance nephrin synthesis, recovering podocin immunofluorescence to 82% of baseline in NZB/W mice (72). Cyclophosphamide diminishes podocyte apoptosis by inhibiting IL-6/JAK2/STAT3 cascades, with combination therapies increasing nephrin-podocin colocalization by 2.3-fold (72). Calcineurin inhibitors (cyclosporine A, tacrolimus) attenuate foot process effacement by obstructing NFAT signaling and facilitating podocin reintegration into slit diaphragms, thus restoring glomerular charge selectivity (73).

### 5.2 Targeted biologics of immune inhibitors in lupus nephritis

Belimumab, a humanized IgG1λ monoclonal antibody, neutralizes soluble BAFF to prevent BAFFR/BCMA/TACI receptors and curtailing pathogenic B-cell expansion and autoantibody generation (74–78). The landmark BLISS-LN phase III trial revealed that belimumab adjunctive therapy significantly enhanced primary and complete renal responses at 104 weeks compared to standard care alone, with a safety profile matching placebo (79, 80). However, the trial’s findings must be interpreted with caution, as its relatively short follow-up duration and underrepresentation of patients with severe proteinuria or class V LN limit generalizability. Efficacy was more pronounced in patients on mycophenolate mofetil (MMF) induction, possibly due to baseline disease heterogeneity (79, 81). While proliferative LN with mild proteinuria showed 9–10% improvements in PERR/CRR, membranous LN or high-proteinuria subgroups derived no significant benefit (82). East Asian populations confirmed its robust efficacy, demonstrating a 63% reduction in renal risk (83), culminating in its 2019 FDA approval as the first SLE-specific biologic.

Telitacicept simultaneously inhibits BAFF and APRIL, disrupting their binding to BCMA/TACI and impairing long-lived plasma cell survival and B-cell hyperactivity (84). A retrospective analysis of 72 active SLE patients (34 with LN) reported median reductions of 77% in 24-hour proteinuria and 75% in anti-dsDNA titers at 24 weeks, with PRR and CRR rates of 76.24% and 70.58%, respectively. By 52 weeks, CRR reached 66.67% (6/9), with infections being the most frequent adverse event (23.6%) (85). Following global phase III trial approvals (NMPA/EMA), large-scale efficacy validation is underway. In refractory LN, sequential telitacicept-rituximab therapy achieved 100% CRR at 19 months, surpassing belimumab monotherapy without increased infection risk (15), suggesting superior efficacy in treatment-resistant cases pending further verification. Rituximab is a chimeric anti-CD20 IgG1κ antibody that depletes CD20^+^ B cells through ADCC and CDC mechanisms while dampening T-cell-driven autoimmunity (86, 87). The LUNAR phase III trial did not meet its primary endpoint, with no significant renal response improvement at 52 weeks in class III/IV LN, potentially due to CDC impairment in hypocomplementemic states and BAFF rebound (88–90). Nevertheless, refractory LN cohorts demonstrate clinical utility: a meta-analysis of 300 patients revealed a 74% overall response rate, with superior outcomes in class III LN (91, 92). Prospective studies corroborate its glucocorticoid-sparing effects and acceptable safety (93), leading to its classification as a second-line refractory LN therapy in the 2024 KDIGO guidelines (94).

### 5.3 Innovative immunomodulatory approaches

The proteasome inhibitor bortezomib induces plasma cell apoptosis via endoplasmic reticulum stress and NF-κB pathway inhibition. In NZB/W F1 mice, bortezomib significantly depleted splenic and bone marrow-resident long-lived plasma cells (95–97). A Japanese multicenter randomized controlled trial in refractory SLE reported a 75% SLE Responder Index at 12 weeks, compared to 40% in placebo, despite similar anti-dsDNA levels at 24 weeks (98). In China, five patients with high-activity LN showed partial renal response after four cycles, with 60% achieving complete renal response (CRR) and 20% progressing to end-stage renal disease over three years (99). Another Spanish retrospective study found that bortezomib reduced median SLEDAI from 27 to 0, with CRR and PRR rates of 8.3% and 83.3%, respectively, though hypogammaglobulinemia (IgG <500 mg/dL) occurred in 50% of patients (100). However, the immunosuppressive side effects of bortezomib require serious attention, with infection being the most frequently observed adverse event (101). These safety concerns, combined with small sample sizes and limited follow-up constrain conclusions regarding long-term efficacy and safety (98–100). Alternatively, daratumumab, a CD38-targeting IgGκ monoclonal antibody, depletes >90% of CD38^+^plasma cells via antibody-dependent cellular cytotoxicity (ADCC) in bone marrow and inflamed tissues (97, 102, 103). In Germany, two refractory LN cases treated with daratumumab-belimumab showed a 50% reduction in anti-dsDNA antibodies and a 62 ± 8% decline in interferon signature gene expression by week 12, with no severe adverse events (102). Similarly, an Italian cohort of six refractory LN patients demonstrated a 66% reduction in SLEDAI, 86% decrease in proteinuria, 35% improvement in serum creatinine, and 1.8-fold rise in C4 after 12 months (104). Daratumumab demonstrates comparable autoantibody clearance efficacy to bortezomib with a trend toward lower infectious adverse events (98, 104, 105), supporting its therapeutic promise in refractory LN.

### 5.4 Natural compounds reprogram TME in lupus nephritis

Macrophages, as a major subset of innate immune cells, represent the predominant infiltrating cell population in the kidneys of LN patients. Compounds targeting macrophage polarization include total glucosides of paeony, which upregulate PD-L2 expression via STAT6 phosphorylation, inducing M2-like macrophage polarization and exerting immunosuppressive effects to attenuate glomerular damage in LN mice (106). Dihydroartemisinin (DHA), a metabolite of artemisinin with potent antimalarial properties, also demonstrates remarkable anti-inflammatory and immunomodulatory activities. By inhibiting ITK signaling to suppress T follicular helper (Tfh) cells and reducing serum levels of IgG, IgM, IgA and anti-dsDNA antibodies, DHA significantly alleviates LN symptoms (107). Genetically engineered macrophage membranes overexpressing CCR2 can enhance the targeted delivery of DHA-loaded nanoparticles to inflammatory sites in LN, thereby reducing monocyte/macrophage infiltration and reprogramming the M1/M2 macrophage balance to modulate the renal immune microenvironment and ameliorate kidney injury (108). Artesunate, a semi-synthetic derivative of artemisinin, improves lupus nephritis symptoms by regulating the T follicular regulatory to T follicular helper cell ratio and activating the JAK2-STAT3 signaling pathway, consequently decreasing renal anti-dsDNA antibody deposition and reducing pathogenic cytokine levels including IL-6, IFN-γ, and IL-21 (109). Furthermore, LLDT-8, a novel triptolide analog, has been demonstrated by ZHANG et al. (28) to exhibit therapeutic effects against LN through suppressing chemokine and IL-6 expression, decreasing renal macrophage and neutrophil infiltration, and reducing glomerular IgG deposition in LN mice (110).

## 6 Conclusion

LN is driven by a multifaceted network of autoimmunity in which podocytes are not merely passive victims but active contributors to renal inflammation and immune dysregulation. As the field progresses, a paradigm shift is emerging that positions podocytes as both biomarkers and therapeutic targets. Advances in our understanding of podocyte-specific signaling, such as VEGF Notch, iNOS autophagy, and cytokine-mediated injury, have catalyzed the development of more refined, histotype-specific interventions. The rise of targeted biologics, including belimumab and telitacicept, marks a turning point in LN management with promising efficacy in modulating B cell and plasma cell function. Novel immunomodulators like bortezomib and daratumumab further expand treatment options, particularly in refractory LN characterized by persistent autoantibody production. These agents also hold the potential to indirectly preserve or restore podocyte function by reducing the burden of circulating immune complexes and complement activation.

Moving forward, integrating urinary podocyte-associated biomarkers into clinical practice may enable real-time disease stratification and therapeutic monitoring. Moreover, therapies aimed directly at stabilizing podocyte architecture, modulating cell-to-cell signaling with glomerular endothelial cells, or enhancing autophagic resilience represent exciting frontiers. Longitudinal studies addressing the durability of remission, infection risks, and population-specific responses to therapy are urgently needed. Ultimately, a podocyte-centered therapeutic framework guided by immunological profiling and biomarker-informed personalization may offer a path toward durable renal protection and reduced reliance on nonspecific immunosuppression in LN.
